# Clinical outcomes and practical management implications in rudimentary horn pregnancies: a systematic review and pooled analysis (2010–2025)

**DOI:** 10.1007/s00404-026-08455-7

**Published:** 2026-05-12

**Authors:** Norah L. A. Emrich, Laura Tascón Padrón, Carolin Schröder, Alexander Mustea, Brigitte Strizek, Ulrich Gembruch, Jorge Jiménez Cruz

**Affiliations:** 1 Department of Obstetrics and Prenatal Medicine, University Hospital Bonn, Bonn, Germany; 2 Department of Gynecology and Gynecological Oncology, University Hospital Bonn, Bonn, Germany; 3 Department of Gynecological Endocrinology and Reproductive Medicine, University Hospital Bonn, Bonn, Germany

**Keywords:** Mullerian anomaly, Uterus malformation, Communicating, Non-communicating, Abortion, Unicornuate uterus, Pregnancy, Uterus rupture

## Abstract

**Purpose:**

Rudimentary horn pregnancies are rare and carry significant maternal and fetal risks. This study aims to systematically review published cases of rudimentary horn pregnancies describing data about time of diagnosis, time of rupture, and live birth rate (LBR), with exploratory comparisons between communicating (CHP) and non-communicating horn pregnancies (NCHP).

**Methods:**

A PRISMA-guided systematic search of PubMed, MEDLINE, and Cochrane (October 1, 2025) was performed with terms “unicornuate uterus pregnancy” OR “rudimentary horn pregnancy.” Case reports, series, and reviews with defined clinical data were included; abstracts and unclear reports were excluded. Three independent reviewers extracted data using a standardized protocol. Descriptive statistics (means, medians, standard deviations, and ranges) and exploratory group comparisons (Fisher's exact test, Mann–Whitney U and Student's t test with *p* < 0.05 denoting significance) were performed.

**Results:**

From 132 articles, 190 cases were included: 27 CHP (14.2%) and 163 NCHP (85.8%). Time of diagnosis was earlier in NCHP (15.9 ± 8.9 weeks) than CHP (22.1 ± 10.0 weeks, *p* = 0.01). Rupture occurred in 68 cases (35.8%), similarly in CHP (48.1%) and NCHP (33.7%, *p* = 0.19). Time of rupture was earlier in NCHP (18.7 ± 6.6 weeks) than CHP (23.0 ± 9.2 weeks, *p* = 0.02). Four cases attempted to continue pregnancy; all resulted in premature rupture. Reported live births were infrequent (11.6%) and more common in published CHP (25.9%) vs. NCHP cases (9.2%; *p* = 0.02).

**Conclusions:**

This descriptive synthesis indicates uterine horn communication may be associated with later diagnosis/rupture and reported live births were significantly more frequent in published CHP vs. NCHP cases. However, all comparisons are exploratory and must be interpreted with extreme caution, as data limitations preclude causal inference. Exhaustive imaging and diagnosis are essential to characterize horn type. Surgical termination is advised, but if expectant management is chosen, close monitoring and multidisciplinary care, including weekly ultrasounds, are advisable to mitigate complications.

**Supplementary Information:**

The online version contains supplementary material available at 10.1007/s00404-026-08455-7.

## Introduction

### Background

A pregnancy in a unicornuate uterus with a rudimentary horn [1] is very rare, with an estimated incidence of 1:76,000–140,000 [2, 3]. The rudimentary horn results from maldevelopment of one Müllerian duct. According to the European Society of Human Reproduction and Embryology (ESHRE)/European Society for Gynaecological Endoscopy (ESGE) consensus classification, unicornuate uterus with rudimentary horn is designated as U4a (hemi-uterus with rudimentary horn), characterized by a unilateral uterine cavity with a contralateral rudimentary horn that may be communicating (cavity connection present) or non-communicating (no cavity connection) and may contain functional endometrium [4–6]. In 72–85% of cases, the two horns are non‑communicating [7–9]. The rudimentary horn may contain functional endometrium or consist only of myometrial tissue [2]. Pregnancy in a blind, non‑communicating horn likely results from transperitoneal migration of sperm or a fertilized ovum and carries a high risk of uterine rupture (50–90%) and maternal mortality (0.5–5.7%) [9–12]. Only 8—14% (of rudimentary horn pregnancies) are diagnosed before symptoms like abdominal pain or signs of rupture occur [13, 14]. Due to severe maternal‑fetal risks and recurrence potential, surgical removal of the pregnant horn is the standard of care [8, 15]. However, some rare reports describe pregnancies reaching peri-viable gestational ages (> 24 weeks) or even live births, typically requiring emergency delivery with significant maternal morbidity and neonatal complications [3, 9, 14, 16–28]. These exceptional cases do not represent normal outcomes but may encourage patients to attempt continuation despite the overall poor prognosis. Therefore, detailed counseling is essential, though it remains challenging because available evidence is limited to case series and reports.

### Objectives

To synthesize evidence coming from published cases from the past 15 years on clinical outcomes in rudimentary horn pregnancies, with the goal of supporting comprehensive patient counseling and clinical decision‑making, focused on pregnancy viability, maternal and fetal morbidity, and risk factors for complications. Primary outcomes were time point of diagnosis (TOD), time point of rupture (TOR), and live birth rate (LBR), with particular emphasis on differences between communicating (CHP) and non‑communicating horn pregnancies (NCHP).

## Methods

### Eligibility Criteria

Case reports, case series, and reviews published in English from 2010 to September 2025 were included. Only articles providing sufficient clinical data on rudimentary horn pregnancies, including gestational age at diagnosis, horn type (communicating vs. non-communicating), maternal and fetal outcomes, and management strategies met the inclusion criteria. Abstracts and letters to the editor, incomplete reports about investigated variables and studies with unclear uterine anomaly definitions were excluded. In reviews and series containing multiple cases, the main report was used as reference independently of the year of publication of each individual case.

### Information sources

This systematic literature review was performed following the recommendations of the PRISMA statement [29]. PubMed, MEDLINE, and Cochrane Library were consulted systematically on October 1, 2025.

### Search strategy

For each database, the MeSH terms and keywords “unicornuate uterus pregnancy” OR “rudimentary horn pregnancy" (all fields) were used. The research was limited from 2010 to September 2025.

### Study selection

Three independent reviewers screened titles and abstracts for eligibility. Full-text articles were obtained and reviewed for the inclusion criteria. Any disagreements regarding inclusion were resolved through discussion with a senior author.

### Data extraction

Data extraction was performed using a standardized table. Extracted variables included gestational age at diagnosis, intention of termination or prolongation of pregnancy, communicating or non-communicating rudimentary horn, rupture and gestational age of rupture, perinatal survival, maternal morbidity and side of the horn. Data on diagnostic methods like documented ultrasound, magnetic resonance imaging (MRI), hysterosalpingography (HSG) or laparotomy / laparoscopy were also recorded. Definition of communication between horns needed interpretation in some cases. Horns connected only by a small fibrous band or lacking a cavity were classified as non-communicating. Two reviewers cross-checked data to ensure accuracy, and discrepancies were resolved by a senior reviewer.

### Assessment of risk of bias

The tool proposed by Murad et al. [30] was used to systematically evaluate selection, ascertainment, causality, and reporting bias across cases. However, risk of bias assessment remains severely limited in case report reviews of ultra-rare conditions like rudimentary horn pregnancy. Selective reporting, missing negative outcomes, and diagnostic verification bias remain unquantifiable and substantially influence the available evidence precluding formal statistical inference [31].

### Outcome definition and statistical analysis

Statistical analysis was performed by using SPSS ver27.0 (SPSS inc. Chicago, IL). Categorical variables (e.g., rupture rates, live birth outcomes) were compared between communicating (CHP) and non-communicating (NCHP) rudimentary horn pregnancies using Fisher’s exact test, given the small sample sizes in certain categories. Mann–Whitney U test was used for the analysis of the continuous or ordinal variables where no normal distribution was observed. For all other continuous variables, the Student’s t test was performed. Descriptive statistics, including means, medians, and ranges, were reported. Analyses were exploratory, and comparative *p *values represent nominal associations and descriptive comparison within the limited study context and should not be interpreted as confirmatory. The results were shown by odds ratio (OR) with a 95% confidence interval (CI) or arithmetic mean and standard mean difference (SMD) as appropriate. Statistical significance was defined as an alpha level < 5% (*p* < 0.05). For visual comparison of timepoint of rupture and timepoint of diagnosis box plot was used.

## Results

### Study selection

**Fig. 1:**
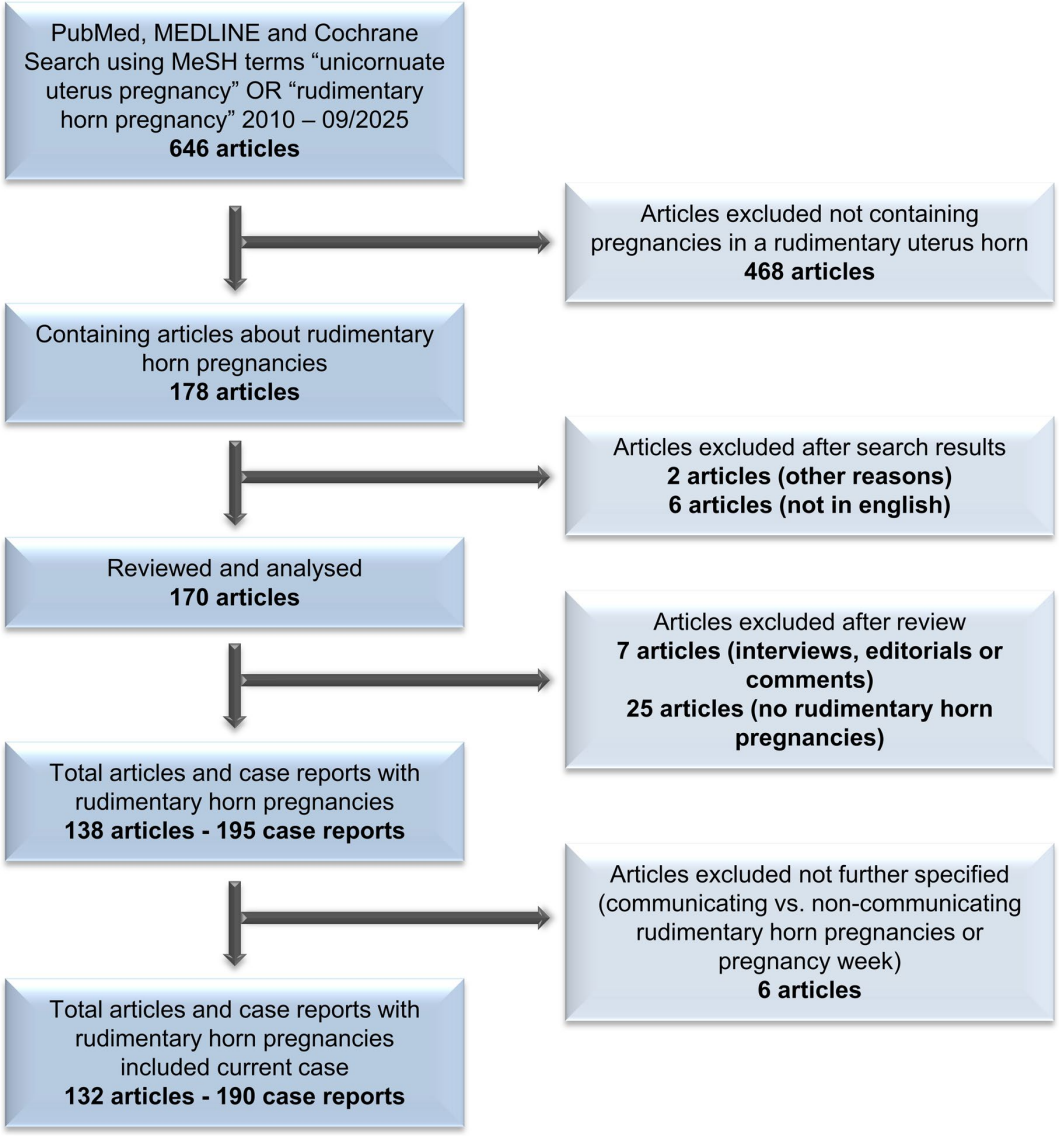
Selection process of included cases

Initial search resulted in 646 articles meeting the MeSH terms, of which 178 articles described a pregnancy in a rudimentary horn. After the review process, 46 manuscripts had to be excluded (reasons given in Fig. 1 and Appendix S1). One current case from our department was included as well (unpublished data). Post hoc sensitivity analysis excluding this case (*n* = 1) yielded materially identical results across all outcomes. Finally, 132 articles were included in the systematic review, assessing data from 190 cases (Fig. 1).

### Synthesis of results

#### Case characteristics

Regarding the characteristics of the uterine malformations, 27 cases (14.2%) were classified as communicating horn pregnancies (CHP) and 163 cases (85.8%) as non-communicating (NCHP). Uterine malformation was known only in 16 cases (8.4%) prior to pregnancy. In one case diagnosis was realized post mortem during the maternal autopsy. A rupture of the rudimentary horn containing the pregnancy occurred in 68 cases (35.8%). From the 4 cases which attempted to carry on pregnancy after diagnosis, all of them ended in rupture of the uterine horn. Live birth has been reported in 22 cases (11.6%). An overview of characteristics of each case can be viewed in Table 1.

**Table 1:** Overview of characteristics of each case

Reference	Case	TOD (weeks)	CHP/NCHP	Side of the horn	Ruptured horn	TOR (weeks)	Live birth	Diagnostic test	Urinary tract anomaly
Dulemba (1996) [34]		13	NCHP	right	no	−	no	US, CT, laparoscopy	no
Dicker (1998) [35]		8	NCHP	right	no	−	no	US, laparoscopy	not evaluated
Yahata (1998) [36]		7	NCHP	right	no	−	no	US, laparoscopy	not evaluated
Yoo (1999) [37]		8	NCHP	right	no	−	no	US, laparotomy	not evaluated
Daskalakis (2002) [33]		7**	NCHP	right	yes	20	no	US, HSG, laparotomy	no
Adolph (2002) [38]		8**	CHP	right	no	−	no	US, laparoscopy	not evaluated
Smolders (2002) [39]		7**	NCHP	left	no	−	no	US, MRI, laparotomy	not evaluated
Edelman (2003) [40]		7	NCHP	right	no	−	no	US, laparoscopy	duplicated right ureter
Chakravarti (2003) [41]		9	NCHP	left	no	−	no	US, laparoscopy	not evaluated
Sutkin (2003) [42]		19	NCHP	right	yes	25	no	US, MRI, laparoscopy, laparotomy	Pelvic right kidney
Ozeren (2004) [43]		17	NCHP	right	no	−	no	US, MRI	no
Cutner (2004) [15]	case 1	12**	NCHP	right	no	−	no	US, laparoscopy	not evaluated
	case 2	12	NCHP	right	no	−	no	US, laparoscopy	not evaluated
Shinohara (2005) [28]		28	NCHP	right	no	28*	yes	US, laparotomy	no
Tsafrir (2005) [44]	case 1	12	NCHP	right	no	−	no	US, laparotomy	no
	case 2	11	NCHP	left	no	−	no	US, HSG	no
Cash (2006) [45]		12	NCHP	right	no	−	no	US	no
Sönmezer (2006) [8]		6**	NCHP	right	no	−	no	US, MRI	no
Goel (2007) [46]		41	NCHP	right	no	42*	yes	US, laparotomy	not evaluated
Park (2007) [47]		8**	NCHP	right	no	−	no	laparoscopy	no
Mavrelos* (*2007) [27]	case 1	13	NCHP	left	no	−	no	laparoscopy	not evaluated
	case 2	13	NCHP	right	no	−	no	US, laparoscopy	not evaluated
	case 3	21	NCHP	left	no	unknown	no	US, HSG, laparoscopy	not evaluated
	case 4	20	NCHP	left	no	unknown	no	US, HSG, MRI, laparoscopy	not evaluated
	case 5	26	NCHP	right	no	32*	yes	US, laparotomy	not evaluated
	case 6	18	NCHP	right	no	unknown	no	US, laparoscopy	not evaluated
	case 7	13	NCHP	right	no	unknown	no	US, laparotomy	not evaluated
	case 8	10**	NCHP	right	no	−	no	US, laparotomy	not evaluated
Sevtap (2007) [48]		6	NCHP	left	no	−	no	US, HSG	not evaluated
Buntugu (2008) [49]		16	NCHP	left	no	−	no	US	no
Contreras (2008) [50]		19	NCHP	left	no	−	no	US	no
Kadan (2008) [51]		11	NCHP	left	no	−	no	US, laparotomy	not evaluated
Henriet (2008) [52]		7	NCHP	left	no	−	no	US, laparotomy	no
Taori (2008) [53]		12	NCHP	left	no	−	no	US, laparotomy	not evaluated
Chopra (2009) [54]	case 1	16	NCHP	right	yes	unknown	no	laparotomy	not evaluated
	case 2	15	NCHP	left	yes	unknown	no	US, MRI	not evaluated
	case 3	24	NCHP	left	no	−	no	US, MRI, laparotomy	not evaluated
	case 4	30	CHP	left	no	−	no	US, CT, laparotomy	not evaluated
	case 5	24	CHP	right	no	−	no	US	not evaluated
	case 6	14	NCHP	left	no	−	no	US	not evaluated
	case 7	12	CHP	right	no	−	no	US	not evaluated
	case 8	15	NCHP	left	yes	unknown	no	US	not evaluated
	case 9	15	NCHP	right	yes	unknown	no	US	not evaluated
	case 10	10	NCHP	left	yes	unknown	no	US	not evaluated
	case 11	34	NCHP	left	no	−	no	US	not evaluated
	case 12	34	NCHP	right	yes	34	no	US	not evaluated
Fitzmaurice (2010) [26]		22	CHP	right	no	25*	yes	US, laparotomy	no
Abreu (2010) [55]	case 1	21	NCHP	left	no	−	no	US, laparoscopy	ipsilateral renal agenesis
	case 2	17	NCHP	left	no	−	no	US, laparotomy	no
Sinha (2010) [23]		32	NCHP	right	no	33*	yes	MRI, laparoscopy, hysteroscopy	not evaluated
van Esch (2010) [56]		10**	NCHP	left	no	−	no	US, laparoscopy, hysteroscopy	not evaluated
Kawthalkar (2011) [57]		37	NCHP	left	yes	34	no	US, laparotomy	no
Matsubara (2011) [58]		20	NCHP	left	yes	20	no	laparotomy	not evaluated
Sharma (2011) [59]		12	NCHP	right	no	−	no	US, MRI	no
Hassan (2011) [60]		19	NCHP	right	yes	19	no	laparotomy	no
Daaloul (2012) [61]		12	NCHP	left	no	−	no	US, laparotomy	not evaluated
Kanagal (2012) [62]		25	NCHP	right	yes	25	no	laparotomy	no
Lim (2012) [63]		10	NCHP	left	no	−	no	laparoscopy	no
Ercan (2012) [64]		10	NCHP	left	no	−	no	HSG, hysteroscopy, laparoscopy	not evaluated
Dhar (2012) [65]		22	NCHP	left	yes	22	no	laparotomy	no
Youssef (2012) [66]		25	CHP	left	yes	25	no	US, laparotomy	no
Thakur (2012) [67]		19	NCHP	left	yes	19	no	US, MRI, laparoscopy	not evaluated
Hirose (2013) [68]		19	CHP	right	yes	19	no	paracentesis, laparotomy	not evaluated
Nathan (2013) [69]		15	NCHP	right	no	−	no	US, laparotomy	no
Gonçalves (2013) [21]		34	NCHP	right	no	34*	yes	US, laparotomy	absent right kidney
Kulkarni (2013) [70]		9	NCHP	right	yes	9	no	laparotomy	not evaluated
Singh (2013) [71]		10	NCHP	left	yes	10	no	US, laparotomy	no
Siwatch (2013) [12]	case 1	21	NCHP	right	yes	21	no	US, laparotomy	not evaluated
	case 2	17	NCHP	right	yes	17	no	US, laparotomy	not evaluated
	case 3	13	NCHP	left	no	−	no	US, laparotomy	not evaluated
	case 4	15	NCHP	left	no	−	no	US, laparotomy	not evaluated
	case 5	24	NCHP	unknown	yes	24	no	US, laparotomy	not evaluated
Lennox (2013) [72]		16	NCHP	left	no	−	no	US, laparoscopy	no
Ambusaidi (2014) [73]		23	NCHP	right	no	−	no	US, MRI, laparotomy	absent right kidney
Fouelifack (2014) [74]		17	NCHP	right	yes	17	no	US	not evaluated
Terzi (2014) [75]		8	NCHP	right	yes	8	no	US	not evaluated
Iyoke (2014) [25]		37	NCHP	left	no	38*	yes	US, MRI, laparotomy	not evaluated
Kanno (2014) [76]		7	CHP	right	no	−	no	US, laparotomy	no
Singh (2015) [77]		20	NCHP	right	no	−	no	US, MRI	not evaluated
Pillai (2015) [19]		32	CHP	left	yes	32	yes	US, MRI, laparoscopy, laparotomy	no
Lallar (2015) [78]		15	NCHP	left	no	−	no	C-section before	not evaluated
Mishra (2015) [79]		16	NCHP	left	yes	16	no	US, MRI, laparotomy	not evaluated
Rathod (2015) [80]		14	NCHP	left	yes	14	no	US, HSG	no
Shrivastava (2015) [24]		27	NCHP	right	no	37*	yes	US	not evaluated
Wang (2015) [81]	case 1	8	NCHP	left	no	−	no	US, laparotomy	not evaluated
	case 2	16	NCHP	left	no	−	no	US, laparoscopy	not evaluated
Cheng (2015) [22]		38	NCHP	right	no	−	yes	US, laparoscopy	not evaluated
Singh (2016) [82]		20	NCHP	left	no	−	no	US, laparotomy	not evaluated
Pannu (2016) [16]		39	CHP	unknown	yes	39	yes	C-section	not evaluated
Lai (2016) [83]		12	NCHP	left	no	−	no	US, laparotomy	not evaluated
Feteh (2016) [84]		43	NCHP	right	no	−	no	autopsy	no
Moawad (2016) [85]		5	NCHP	right	no	−	no	laparotomy, US	no
Juneja (2017) [86]		29	NCHP	right	yes	29	no	US, CT, laparotomy	no
Souza (2017) [87]		45	NCHP	right	no	45	no	C-section	not evaluated
Bodur (2017) [88]		39	CHP	left	no	39*	yes	C-section	not evaluated
Al Qarni (2017) [3]		31	NCHP	left	yes	31	yes	laparotomy	not evaluated
Yildirim (2017) [89]		12	NCHP	right	no	−	no	US, MRI, laparotomy	no
Abd El-Halim (2017) [90]		16	NCHP	right	yes	16	no	US, MRI, laparotomy	not evaluated
Blancafort (2017) [91]		8	NCHP	left	no	−	no	HSG, laparoscopy	no
Kumar (2018) [92]		17	NCHP	right	yes	17	no	US, laparoscopy, laparotomy	not evaluated
Kaveh (2018) [93]		14	NCHP	right	yes	14	no	US, laparotomy	no
Herchelroath (2018) [94]		8	NCHP	right	no	−	no	US, laparoscopy	not evaluated
Hussain (2018) [95]		17	NCHP	right	yes	17	no	laparotomy	no
Brady (2018) [96]		12	NCHP	left	yes	17	no	US, laparotomy	no
Dove (2018) [97]	case 1	9	NCHP	left	no	−	no	US, laparoscopy, laparotomy	not evaluated
	case 2	6**	NCHP	left	no	−	no	US, laparoscopy	left kidney showed a duplicated collecting system
Harzallah (2018) [98]		16	NCHP	right	yes	16	no	C-section before	not evaluated
Tolani (2018) [99]		5	NCHP	right	no	−	no	MRI, laparoscopy	not evaluated
Thurber (2018) [100]	case 1	7**	CHP	links	no	−	no	US	duplicated collecting system ipsilateral
	case 2	12	CHP	links	no	−	no	US	no
	case 3	20	CHP	right	no	−	no	US, MRI	not evaluated
Sánchez-Ferrer (2018) [101]	case 1	9	NCHP	left	yes	9	no	US, laparoscopy	no
	case 3	20	CHP	unknown	yes	20	no	US, laparotomy	no
	case 4	12	NCHP	unknown	yes	12	no	US, laparotomy	no
	case 5	8	NCHP	unknown	no	−	no	US, HSG, laparotomy	no
Li (2019) [17]	case 1	17	NCHP	unknown	yes	17	no	US, laparotomy	no
	case 2	8**	NCHP	unknown	no	−	no	US, laparotomy	not evaluated
	case 3	14	NCHP	unknown	yes	14	no	US, laparotomy	no
	case 4	7	NCHP	unknown	no	−	no	US, laparotomy	no
	case 5	10	NCHP	unknown	no	−	no	US, laparotomy	not evaluated
	case 6	23	CHP	unknown	yes	23	no	US, laparotomy	no
	case 7	14	NCHP	unknown	yes	14	no	US, laparotomy	not evaluated
	case 8	33	CHP	unknown	yes	33	yes	US, laparotomy	no
	case 9	24	CHP	unknown	yes	24	no	US, laparotomy	ipsilateral renal agenesis
	case 10	10	CHP	unknown	yes	10	no	US, laparotomy	not evaluated
	case 11	9	NCHP	unknown	no	−	no	US, laparotomy	ipsilateral renal agenesis
Tesemma (2019) [102]		16	NCHP	left	yes	16	no	US, laparotomy	no
Yassin (2019) [18]	case 1	11	NCHP	right	no	−	no	US, laparoscopy	not evaluated
	case 2	38	NCHP	right	yes	unknown	yes	C-section	not evaluated
Parveen (2019) [103]		20	NCHP	unknown	yes	20	no	laparotomy	not evaluated
Cobec (2019) [104]		7	NCHP	right	no	−	no	US, laparoscopy	no
Monacci (2019) [105]		5	NCHP	right	no	−	no	US, laparotomy	no
Abbasi (2019) [106]		17	NCHP	right	yes	17	no	US	not evaluated
Della Corte (2019) [107]		20	CHP	left	no	−	no	US, laparoscopy	no
Hafizi (2019) [108]		12	NCHP	left	yes	12	no	US, laparotomy	not evaluated
Rodrigues (2019) [109]		6	NCHP	right	no	−	no	US, HSG, laparoscopy	no
Amer (2020) [110]		20	NCHP	unknown	yes	20	no	US, laparotomy	not evaluated
Walker (2020) [111]		8	NCHP	left	no	−	no	US, laparotomy	no
Mengistu (2020) [112]		30	CHP	right	no	−	no	US, laparotomy	no
Zhang (2020) [13]		38**	CHP	right	no	38*	yes	US, laparotomy	no
Ağaçayak (2020) [113]	case 1	16	CHP	left	yes	16	no	US, laparotomy	not evaluated
	case 2	19	NCHP	left	yes	19	no	US, laparotomy	not evaluated
	case 3	14	NCHP	left	no	−	no	US, laparotomy	not evaluated
	case 4	17	NCHP	right	yes	17	no	US, laparotomy	not evaluated
	case 5	8	NCHP	left	no	−	no	US, laparotomy	not evaluated
	case 6	22	NCHP	left	yes	22	no	US, laparotomy	not evaluated
	case 7	13	NCHP	right	no	−	no	US, laparotomy	not evaluated
	case 8	9	NCHP	left	no	−	no	US, laparotomy	not evaluated
	case 9	9	NCHP	right	no	−	no	US, laparotomy	not evaluated
	case 10	16	NCHP	left	no	−	no	US, laparotomy	not evaluated
	case 11	9	NCHP	left	no	−	no	US, laparotomy	not evaluated
	case 12	38	NCHP	left	no	−	no	US, laparotomy	not evaluated
	case 13	30	NCHP	right	no	−	no	US, laparotomy	not evaluated
	case 14	25	NCHP	right	no	−	no	US, laparotomy	not evaluated
Bruand (2020) [114]		12	NCHP	right	yes	12	no	US, laparoscopy, laparotomy	not evaluated
Ross (2020) [11]		12	NCHP	right	no	−	no	US, MRT, laparotomy	not evaluated
Jomaa (2021) [115]		16	NCHP	unknown	no	−	no	US, laparotomy	no
Kozar (2021) [116]		14	NCHP	right	yes	14	no	US, laparotomy	not evaluated
Anwari (2021) [117]		13	CHP	right	yes	13	no	US, laparotomy	not evaluated
Houmaid (2021) [118]		16	NCHP	right	yes	16	no	US, laparotomy	no
Dhanawat (2021) [119]		7	NCHP	right	no	−	no	US, laparoscopy	not evaluated
Ghotra (2021) [120]		12	CHP	right	yes	12	no	US, laparotomy	no
Nelson (2022) [121]		16	NCHP	left	no	−	no	US, MRI, laparotomy	no
Sarikaya (2022) [122]		26	NCHP	left	yes	26	yes	US, MRI, laparotomy	no
Ma (2022) [123]		8	NCHP	right	no	−	no	US, CT, laparoscopy	not evaluated
Isono (2022) [124]		7	NCHP	right	no	−	no	US, MRI, laparoscopy	not evaluated
Jiang (2022) [125]		7	NCHP	right	no	−	no	US, MRI, laparoscopy	not evaluated
Alrawashdeh (2022) [126]		10	NCHP	left	no	−	no	US, MRI, laparoscopy	no
Shamase (2022) [127]		24	NCHP	left	yes	24	no	autopsy	not evaluated
Ekpe (2022) [128]		7**	NCHP	right	no	−	no	US, laparoscopy	no
Wang (2022) [129]		17	NCHP	unknown	yes	17	no	US, CT, laparotomy	not evaluated
Dadgar (2023) [130]		32	NHCP	unknown	yes	32	no	US, laparotomy	not evaluated
Bidiga (2023) [131]		28	CHP	left	no	28*	yes	US, laparotomy	not evaluated
Gunjan (2023) [132]		22	NCHP	right	yes	22	no	US, laparotomy	ectopic malrotated right kidney
Mohammed (2024) [133]		18	NCHP	right	no	−	no	US, laparotomy	not evaluated
Taifur (2024) [134]		30	NCHP	left	yes	35*	yes	US, laparotomy	not evaluated
Ji (2024) [135]		7	NCHP	left	no	−	no	US, MRI, laparoscopy	no
Elito Júnior (2024) [136]		28	NCHP	right	no	34*	yes	US, MRI, laparotomy	not evaluated
Shin (2024) [137]		5**	NCHP	right	no	−	no	US, laparoscopy	not evaluated
Solomon (2024)[138]		33	CHP	right	yes	33	no	US, laparotomy	not evaluated
Yadav (2024)[139]		17	NCHP	left	yes	17	no	US, laparotomy	no
Krishnan (2024)[140]		6**	NCHP	right	no	−	no	US, MRI, laparoscopy	right renal agenesis
Al Abbas (2024)[141]		18	NCHP	left	yes	18	no	US, MRT, laparotomy	not evaluated
Ponniah (2024)[142]		10	NCHP	right	no	−	no	US, laparoscopy	not evaluated
Harpey 2024 [143]		8**	NCHP	right	no	−	no	US, MRI	not evaluated
Rivera Casul (2025) [144]		6	NCHP	right	no	−	no	US, MRI, laparoscopy	not evaluated
Essebbagh (2025) [145]		9	NCHP	left	no	−	no	US, laparoscopy	no
Peters (2025)[146]		10	NCHP	right	no	−	no	US, MRI, laparoscopy,	not evaluated
current case (unpublished data)		12	NCHP	right	yes	16	no	US, MRI, laparoscopy, laparotomy	

^*^asterisk indicating not time point of rupture but gestational age at time point of birth

^**^double asterisk indicates diagnosis of rudimentary uterine horn established prior to pregnancy

*GA* gestational age, *CHP* communicating horn pregnancy, *NCHP* non-communicating horn pregnancy, *US* ultrasound, *HSG* hysterosalpingography, *MRI* magnet resonance imaging, *CT* computed tomography, *TOD* time point of diagnosis, *TOR* time point of rupture

Concerning the side of the uterine horn, in 21 cases (11.1%), no side of the blind horn was described. Of the remaining cases, 94 cases (49.5%) were localized right-sided and 75 cases (39.5%) left sided (*p* > 0.05).

The standard diagnostic test was ultrasound; it was performed in 167 cases (87.9%). MRI was used in only 31 cases (16.3%). A diagnostic / therapeutic laparoscopy was accomplished in 127 (66.8%) of all cases. In 37 cases (19.5%) an emergency laparotomy was needed (Table 2).

**Table 2:** Differences between pregnancy outcomes of NCHP vs CHP

	NCHP (%) 163 (85.8)	CHP (%) 27 (14.2)	Total (%) 190 (100)	*p*-value
Gestational age at diagnosis (mean ± SD)	15.9 ± 8.9 wk	22.1 ± 10.0 wk	16.7 ± 9.3 wk	< 0.01*
Gestational age at rupture (mean ± SD)	18.7 ± 6.6 wk	23.0 ± 9.2 wk	19.5 ± 7.2 wk	0.02*
Rupture of the horn	55 (33.7)	13 (48.1)	68 (37.0)	0.19
Live birth	15 (9.2)	7 (25.9)	22 (11.6)	0.02*
Known before pregnancy	13 (8.0)	3 (11.1)	16 (8.4)	0.71
Diagnostic laparoscopy	118 (72.4)	9 (33.3)	127 (66.8)	< 0.01*
Emergency laparotomy	28 (17.2)	9 (33.3)	37 (19.5)	0.07
Maternal death	2 (1.6)	1 (3.7)	3 (1.6)	0.37
Erythrocyte concentrates	32 (19.6)	4 (14.8)	36 (18.9)	0.74
Horn side distribution	Right: 48.9% (84/172) Left: 38.4% (66/172)	Right: 55.6% (10/18) Left: 44.4% (8/18)	Right: 49.5% (94/169)	0.05

^*^statistically significant (p < *0.05)*

Regarding additional malformations of the urinary tract, which are commonly associated to uterine malformations, only in 79 cases urinary tract malformations were evaluated, those being present only in 13 cases (16.4% of the cases where malformations were evaluated at all). The most common associated malformations were unilateral renal agenesis (7 cases), ectopic kidney (2 cases), and duplication of renal structures (4 cases).

#### Differences between NCHP and CHP

The mean gestational age at diagnosis was in the group of NCHP 15.9 weeks (± 8.9) and in the group of CHP 22.1 weeks (± 10.0), with this difference being statistically significant (*p* = 0.01). A rupture of the rudimentary horn was registered more often in CHP with 13 cases (48.1%) than in NCHP with 55 cases (33.7%), but this difference was not statistically significant (*p* = 0.19, Tables 1 and 2). TOR was significantly earlier in NCHP than in CHP (18.7 ± 6.6 vs. 23.0 ± 9.2 weeks, *p* = 0.02, Table 2).

#### Surgical interventions and maternal morbidity

Laparoscopy was performed in 127/190 cases (66.8%) primarily for therapeutic/diagnostic purposes. In patients consenting to pregnancy termination, laparoscopic resection of the rudimentary horn and ipsilateral fallopian tube was completed. All laparoscopic resections succeeded when performed pre-rupture; this approach offers lower morbidity compared to laparotomy when expertise is available. Emergency laparotomy was required in 37/190 cases (19.5%) due to rupture. Maternal death occurred in 3/190 cases (1.6%), all following rupture of non-communicating rudimentary horns. Transfusion of erythrocyte concentrates was needed in 36/190 cases (18.9%), with rupture identified as the sole significant predictor (OR 39.9, 95% CI 11.4—139). Clinical presentation of rupture typically included acute abdominal pain (most common), hemoperitoneum, and maternal hypovolemia. Hysterectomy was performed in only two cases (1.1%). Among all 190 cases, live birth occurred in 7/68 ruptured pregnancies (10.4%) versus 15/122 non-ruptured pregnancies (12.3%; *p* = 0.82), indicating rupture was not significantly associated with live birth rate.

The reported live birth rate was infrequent overall (11.6%) and was significantly different depending on communication between horns (*p* = 0.02). This being 15 cases (9.2%) in the subgroup of NCHP and seven cases (25.9%) in the subgroup of CHP (Table 2). As expected, when a rupture occurred, TOR was significantly later in the live birth group (33.3 (± 4.4) weeks vs. 17.6 (± 5.4) weeks, respectively (*p* < 0.01)).

## Discussion

### Main findings and interpretation

This literature review highlights the high complexity of counseling and management of a pregnancy in a rudimentary horn, particularly when patients wish to continue the pregnancy.

To the best of our knowledge, this is the first systematic review of rudimentary horn pregnancies since Siwatch et al. (2013; *n* = 26 cases, 2001–2010), which analyzed data over 15 years ago [12]. Another extensive review was published 2002, containing 588 published cases from 1900 to 1999 [9]. The present review means to update these reviews including literature from the past 15 years.

As shown in this review the most common complication of this form of ectopic pregnancies is the rupture of the horn, this being a potentially life-threatening course and associated with high maternal morbidity [9, 12]. Operative termination with resection of the gravid rudimentary horn remains the conventional approach to avert rupture risk and recurrence. While published case reports describe occasional live births, these exceptional outcomes—prone to publication and survivorship bias—do not support recommending pregnancy continuation.

Achieving an accurate diagnosis regarding location, communication of the horns and description of the malformation is challenging on regular gynecologic examination and gestational age of rupture seems to be unpredictable, so no clear monitoring strategies have been described so far for those women rejecting termination of pregnancy.

In the case series of Li et al., only one in eleven referred cases of rudimentary horn pregnancies was diagnosed correctly. The remaining 10 received a misdiagnosis, such as extrauterine pregnancy, uterus didelphys, gestational trophoblastic disease, and appendicitis [17]. Also Sönmezer et al. reports of only 5 of 8 preoperative correct diagnosed rudimentary horn pregnancies, the others were thought to be ectopic, cornual or isthmic pregnancies [8]. Especially if there is no current pain or history of dysmenorrhea, lower abdominal discomfort or cryptomenorrhea, as expected in patients with a blind non-communicating horn, diagnosis can be easily overlooked on routine exploration. MRI is recommended when ultrasound findings are inconclusive regarding horn communication, precise pregnancy location, or myometrial invasion. In this study, MRI was utilized in 16.3% and confirmed diagnosis pre-rupture in complex presentations where ultrasound characterization was limited. In non-pregnant patients, evaluation of uterine malformations should follow the Thessaloniki ESHRE/ESGE consensus diagnostic work-up for female genital anomalies, incorporating 2D/3D ultrasound, hysteroscopy, and MRI as appropriate to standardize classification and guide management [32]. But even prior knowledge about the malformation does not prevent a complicated course [33]. Interestingly, a blind uterus horn seems to occur more frequently on the right side, as Sönmezer et al. described in 7 out of 8 times [8] and Nahum et al. found in 54% of all cases [9]. This trend could also be observed in the present pooled analysis, where 49.5% of the pregnancies occurred in the right-sided rudimentary horn. The cause is presumed to lie in the embryological development of the left Mullerian duct which is developing slightly ahead of the right one [9].

According to data of this literature research, the most common time point of rupture of the blind horn is the second trimester, where 70.6% of all pregnant rudimentary uterine horn ruptures occurred. During the first trimester 14.7% ruptured and 14.7% during the third trimester. These are similar results to Nahum et al. describing 67% ruptures in the second trimester, 13% in the first and 20% in the third trimester [9].

### Comparison with previous reviews

Especially in consideration of fast developing medical features, this group assumes that maternal and fetal mortality rate nowadays are likely to differ from those reported from 1900 to 1999 [9]. During this time, a drastic reduction of maternal death occurred. The study group about Nahum et al. already described a reduction of mortality within time in their analysis 20 years ago, showing an overall maternal mortality of 5.1% from 1900 until 2000 with a peak of 23% during the 1910’s [9]. This, compared to only 1.6% in the current investigation from 2010 until 2025, might show a trend to less maternal deaths, most likely to be explained by the improvement of medical care.

Moreover, fetal survival rate seems to increase in publications of the last years, even if rupture happens. According to Nahum et al., a live birth in > 24 weeks was described in 6.5% of the cases [9]. In the current investigation, 22 cases (11.6%) of all rudimentary horn pregnancies resulted in a live birth. In the present review, rudimentary horn rupture was also associated with a reduction of fetal survival. Furthermore, the medical care has remarkably improved in the last years increasing survival of premature fetuses at early gestational age.

### Implications of accessory horn’s anatomy

Present data suggest that pregnant women with a CHP had a better maternal and fetal outcome than those with a NCHP. The observed earlier rupture tendency for NCHP could be the underlying cause, which could also explain the earlier diagnostic time point in this group. However, due to data limitations, findings should be interpreted cautiously, as later gestations and live births are more likely to be reported; they represent described outcomes, not true incidence or safety data.

### Limitations

This study may present some noteworthy limitations. Due to the rarity of this diagnosis only small case series and case reports have been published, so the number included in this review remains small. In the same line, reliance on published case reports introduces potential biases, including selective reporting and data heterogeneity. The lack of variance measures limits generalizability and formal statistical inference. The presented comparative pooled analysis has only descriptive intention and should be considered with care, since data sources are very heterogenous. Regarding the diagnostic pathways, no standardization was present across reports and imaging sensitivity and availability varied widely, being ultrasound the predominant imaging modality.

### Proposal for patient management—flow chart

**Fig. 2:**
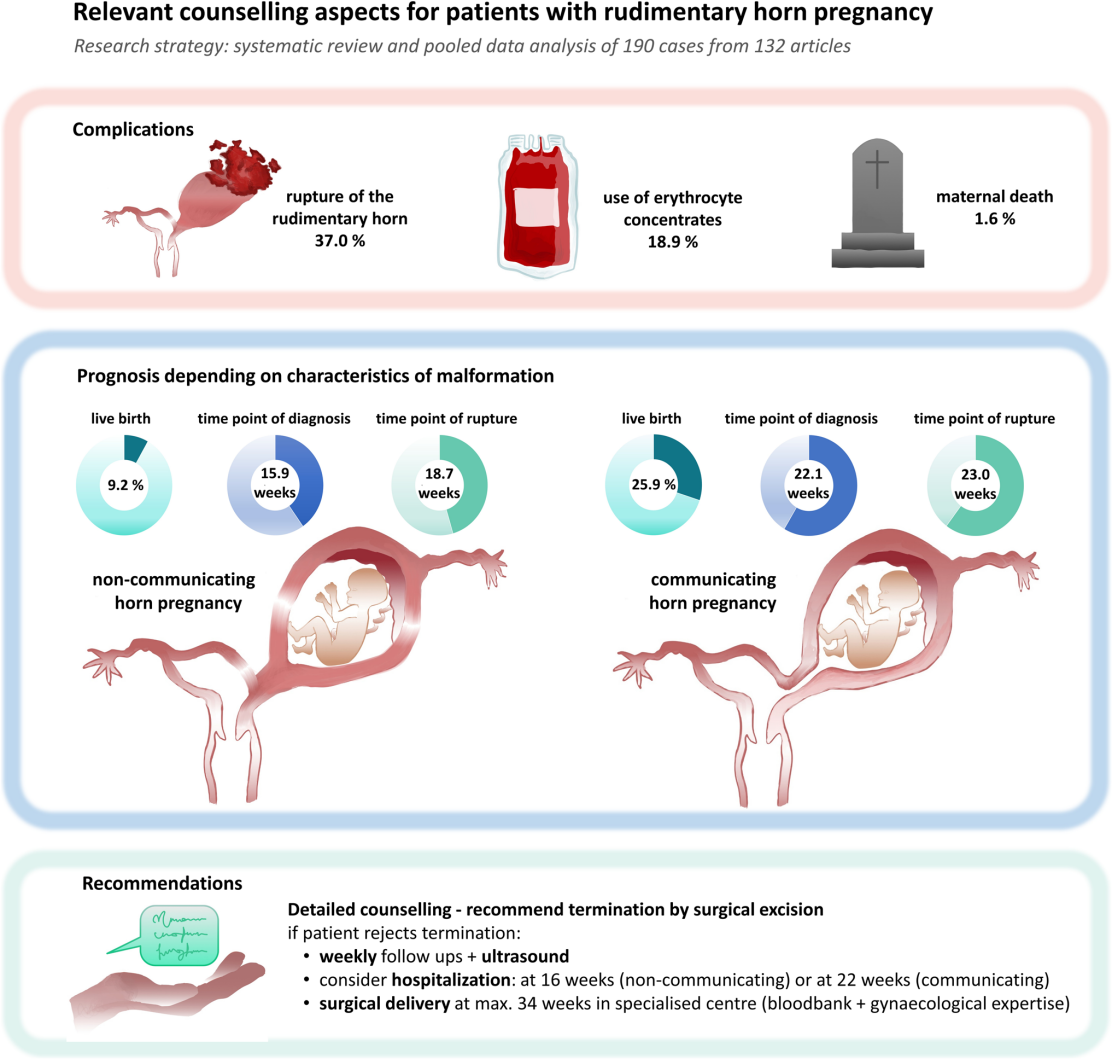
Graphical support for patient counseling regarding rudimentary horn pregnancy

**Fig. 3:**
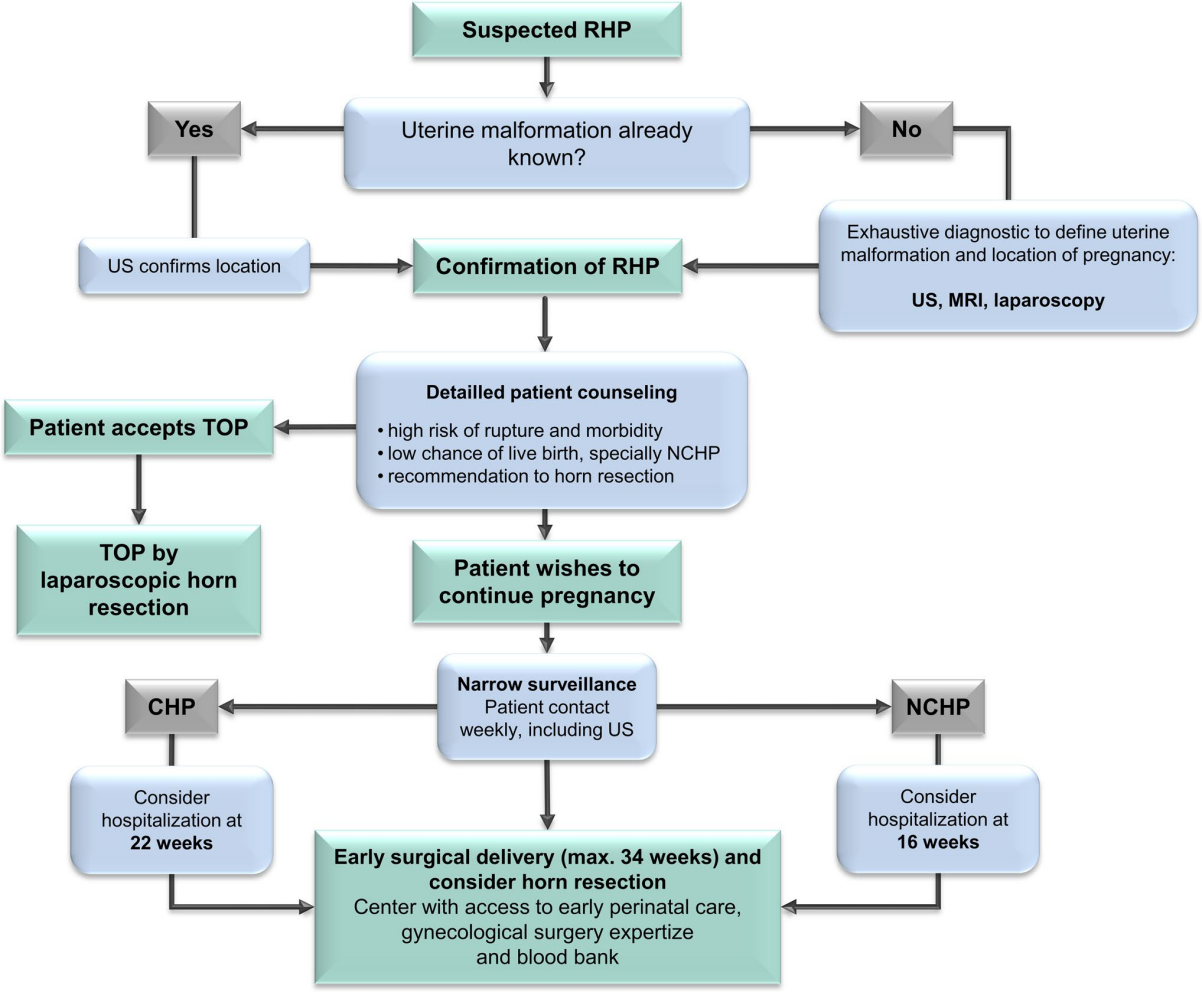
Flow chart—proposal of managing pregnancies with RHP. *US* ultrasound, *MRI* magnetic resonance imaging, *TOP* termination of pregnancy, *CHP* communicating horn pregnancy, *NCHP* non-communicating horn pregnancy

Based on our experience and the data presented in this review, we have summarized some recommendations that, in our opinion, could contribute to improving patient management. The following represents cautious expert opinion derived from low-level evidence (case reports prone to bias) and should support individualized shared decision-making, not prescriptive guidance. A high effort should be performed to evaluate the location of the pregnancy. A diagnostic work-up is recommended, including sonography and, if indicated, an MRI scan and diagnostic laparoscopy, in order to correctly describe the uterine malformation, to assess the location of the pregnancy and to eventually identify a communication between the two horns. Based on this information, patient counseling should be performed exposing all therapeutic possibilities. In particular, detailed counseling is essential regarding the high risk of uterine rupture and the associated maternal morbidity and mortality, as well as the low likelihood of live birth and the need for preterm delivery in cases of ongoing pregnancy. Obtaining informed patient consent is mandatory. As the morbidity and mortality rates of these rudimentary horn pregnancies are very high and the probability of a live birth remains relatively low, the primary recommendation should be the operative extirpation of the malformed horn containing the pregnancy. A graphical support for patient counseling can be found in this manuscript (Fig. 2). If the patient attempts to carry on pregnancy, very narrow surveillance and monitoring should be scheduled and early hospitalization should be considered. Depending on the presence of CHP or NCHP, the time point of recommended hospitalization could vary. For CHP, we recommend hospitalization at 22 weeks’ gestation, while for NCHP, hospitalization is advised from 16 weeks onward. Since achievement of term is rare, optimal gestational age of early surgical delivery (around 34 weeks based on benefit–risk assessment) should be planned and performed in a center with both expertise for premature delivery and complex gynecological surgery with access to blood bank, since transfusion of erythrocyte concentrates are needed in almost 20% of all cases. Furthermore, serial obstetric sonography could help in management of these pregnancies, not only for the evaluation of fetal development and for fetal surveillance, but also for monitoring wall thickness of the blind horn (Fig. 3).

Routine renal imaging is recommended given the 16.4% anomaly rate in evaluable cases (vs. > 30% literature baseline) [3, 9] though inconsistent reporting limits precise prevalence estimates. A prerequisite for that individual approach is a compliant patient being able to estimate the given risk.

This review and clinical experience support management recommendations based on low‑level case report evidence, warranting cautious and site‑specific application.

## Conclusion

Pregnancy in the rudimentary horn of a unicornuate uterus is a rare situation with a very poor prognosis for the fetus and potentially life-threatening status for the mother. The data presented in this review point out that the characteristics of the uterine malformation may influence the clinical course. Exhaustive diagnostic work-up could help to describe these characteristics like communication of both uterine horns, since CHP seem to have a better outcome compared to NCHP. Despite high morbidity, some patients could decide not to terminate pregnancy. In the case of expectant management, individual close monitoring and therapeutic plan conducted by a multidisciplinary team should be performed in order to reduce complications.

## Supplementary Information

Below is the link to the electronic supplementary material.

Supplementary file1 (DOCX 106 KB)

Supplementary file2 (DOCX 32 KB)

## Data Availability

The data underlying this article are available in the article and in its online supplementary material.
