# Supplementary material for: Clinical outcomes and practical management implications in rudimentary horn pregnancies: a systematic review and pooled analysis (2010–2025)
Source: Arch Gynecol Obstet. 2026 May 12;313(1):194. doi: 10.1007/s00404-026-08455-7 (PMC13167825; doi:10.1007/s00404-026-08455-7)
Supplement: Supplementary file 1 — Supplementary file1 (DOCX 106 KB) [file 404_2026_8455_MOESM1_ESM.docx]

**Appendix S1** Excluded papers

| **Reference** | **Case** | **Title** | **Reason for exclusion** |
| --- | --- | --- | --- |
| **Jain (2010)^1^** |  | A rare case of intact rudimentary horn pregnancy presenting as hemoperitoneum | Uterus didelphys, no rudimentary horn |
| **Wang (2011)^2^** |  | Torsion of a rudimentary uterine horn at 22 weeks of gestation | Intrauterine pregnancy in unicornatus uterus, not in rudimentary horn |
| **Allouche (2011)^3^** |  | Grossesse dans une corne utérine rudimentaire: une cause rare d’hémopéritoine spontané au deuxième trimestre de grossesse | Not in English |
| **Siristatidis (2011)^4^** |  | Rudimentary-horn pregnancy: some points to review | Letter to the editor |
| **Brucker (2011)^5^** |  | Treatment of Congenital Malformations | Patients not pregnant |
| **Khati (2011)^6^** |  | The Unicornuate Uterus and Its Variants | Patients not pregnant |
| **Nawfal (2011)^7^** |  | Laparoscopic Management of Pregnancy in a Patient with Uterus Didelphys, Obstructed Hemivagina, and Ipsilateral Renal Agenesis | Pregnancy in unicornuate uterus, not in rudimentary horn |
| **Mathlouthi (2013)^8^** |  | Grossesse sur corne utérine rudimentaire | Not in English, letter to the editor |
| **Köroğlu (2013)^9^** |  | MR imaging of ectopic pregnancy with an emphasis on unusual implantation sites | No rudimentary horn pregnancy |
| **Lata (2013)^10^** |  | Ruptured rudimentary horn pregnancy misdiagnosed as ruptured pseudo aneurysm internal iliac artery | Letter to the editor |
| **Munck (2013)^11^** |  | Pregnancy in a non-communicating rudimentary uterine horn in an obese woman | Letter to the editor |
| **Torbe (2013)^12^** |  | Subsequent pregnancy after a ruptured rudimentary uterine horn pregnancy | Letter to the editor |
| **Alkatout (2013)^13^** |  | Clinical Diagnosis and Treatment of Ectopic Pregnancy | Comment, no case report |
| **Caserta (2014)^14^** |  | Pregnancy in a unicornuate uterus: a case report | Pregnancy in unicornuate uterus, not in rudimentary horn |
| **Akdemir (2014)^15^** |  | Coring-type laparoscopic resection of a cavitated non-communicating rudimentary horn under hysteroscopic assistance | Patient not pregnant |
| **Pados (2014)^16^** |  | Reproductive and obstetric outcome after laparoscopic excision of functional, non-communicating broadly attached rudimentary horn: a case series | Case series (8 cases) of pregnancies/fertility rate after excision of a rudimentary horn |
| **Arab (2014)^17^** |  | Functional Rudimentary Horn as a Rare Cause of Pelvic Pain: A Case Report | Case report about diagnosis of rudimentary horn, no pregnancy in rudimentary horn |
| **Takeda (2015)^18^** |  | Magnetic resonance imaging for diagnosis and management of unruptured interstitial pregnancy | Interstitial pregnancy, not in rudimentary horn |
| **Aheri (2015)^19^** |  | La grossesse gémellaire sur un utérus pseudo unicorne: à propos d’un cas | Not in English |
| **Shiber (2015)^20^** |  | Laparoscopic Management of a Rudimentary Horn Pregnancy | Comment, no case report |
| **Dibble (2015)^21^** |  | Imaging Unusual Pregnancy Implantations: Rare Ectopic Pregnancies and More | Comment, no case report |
| **Le Mitouard (2016)^22^** |  | Rupture utérine colmatée par l’épiploon sur grossesse développée au dépend d’une corne utérine rudimentaire: à propos d’un cas rare | Not in English |
| **Gnanasambanthan**  **(2017)^23^** |  | A rare case of an ectopic pregnancy in the rudimentary tube of a patient with an isolated unicornuate uterus | Tubal pregnancy, no pregnancy in rudimentary horn |
| **Rutten (2017)^24^** |  | Lithopedion developed in a non-communicating rudimentary uterine horn: CT features | Lithopedion (stone child) in rudimentary uterine horn - tolerated for 18 years |
| **Bayyarapu (2017)^25^** |  | Diagnosis and Management of 'Cornual' Pregnancies from 2002 to 2015 at a Tertiary Referral Centre in South India: Insights from Introspection | Not further specified (communicating vs. non-communicating rudimentary horn pregnancy) |
| **Ngichabe (2017)^26^** |  | Placenta Percreta in a Gravid Bicornuate Unicollis Uterus | Bicornuate uterus, no rudimentary horn pregnancy |
| **Si (2017)^27^** |  | An unexpected invasive hydatidiform mole in a rudimentary uterine horn: A case report | Invasive mole in a rudimentary uterine horn |
| **Sánchez-Ferrer (2018)^28^** | 2 | Variations in clinical presentation of unicornuate uterus with non-communicating rudimentary horn (class IIB of the American Fertility Society classification) | Patient not pregnant |
| **Zhang (2018)^29^** |  | Diagnosis and Treatment of Rudimentary Horn Pregnancy: Analysis of Eleven Cases | Incongruent in contiunous text and tables |
| **Sawada (2018)^30^** |  | Obstetric outcome in patients with a unicornuate uterus after laparoscopic resection of a rudimentary horn | Pregnancy in unicornuate uterus, not in rudimentary horn |
| **Obeidat (2019)^31^** |  | Unicornuate uterus with a rudimentary non-communicating cavitary horn in association with VACTERL association: case report | Patient not pregnant |
| **Engku-Husna (2020)^32^** |  | Müllerian duct anomalies with term  pregnancy: a case report | Pregnancy in unicornuate uterus, not in rudimentary horn |
| **Schaible (2021)^33^** |  | Abdominal cerclage in a patient with a neocervix with planned cesarean hysterectomy at delivery | Pregnancy in unicornuate uterus, not in rudimentary horn |
| **Ebanga (2021)^34^** |  | Original two steps management of an ectopic pregnancy on rudimentary horn in a patient with an unicornuate uterus: A unique case report with a literature review | Not in English |
| **Coakley (2021)^35^** |  | Periviable delivery of a pregnancy in a rudimentary uterine horn: A case report | Not further specified (communicating vs. non-communicating rudimentary horn pregnancy) |
| **Yagmurov (2022)^36^** |  | Problems of expert assessment of a case of fatal rupture of the wall of the rudimentary horn of the uterus in ectopic pregnancy | Not in English |
| **Hamet (2022)^37^** |  | Pregnancy in a rudimentary horn: multicenter's MRI features of a rare condition | Not further specified (communicating vs. non-communicating rudimentary horn pregnancy) |
| **Tellum (2023)^38^** |  | Reproductive outcome in 326 women with unicornuate uterus | Not further specified (communicating vs. non-communicating rudimentary horn pregnancy) |
| **Aminu (2023)^39^** |  | Post-dated Breech Pregnancy in a Non-obviously Communicating Rudimentary Horn of a Bicornuate Uterus Requiring Hemi-hysterectomy | Pregnancy in bicornuate uterus, not in rudimentary horn |
| **Zheng (2023)^40^** |  | Abdominal pregnancy secondary to uterine horn pregnancy: a case report | Intraperitoneal ectopic pregnancy, no rudimentary horn pregnancy |
| **Garapati (2023)^41^** |  | Unicornuate Uterus with a Non-Communicating Rudimentary Horn: Challenges and Management of a Rare Pregnancy | Pregnancy in unicornuate uterus, not in rudimentary horn |
| **Shrivastava (2023)^42^** |  | Rare and Occult Müllerian Deformity Diagnosed Intraoperatively During Lower Segment Caesarean Section | Pregnancy in unicornuate uterus, not in rudimentary horn |
| **Xu (2023)^43^** |  | A case of rudimentary uterine horn pregnancy complicated with placental implantation in later stages of pregnancy | Pregnancy in unicornuate uterus, not in rudimentary horn |
| **Srinivas (2024)^44^** |  | Laparoscopic Management of a 12-Week Pregnancy Loss in a Rudimentary Uterine Horn | Not further specified (communicating vs. non-communicating rudimentary horn pregnancy) |
| **Tsai (2024)^45^** |  | Surgical management of an ectopic pregnancy in the setting of an unexpected Müllerian anomaly: intraoperative and postoperative implications | Pregnancy week not specified |
| **Khan (2024)^46^** |  | Clinical profile and management of non-tubal ectopic pregnancy: Experience from a tertiary care hospital in the United Arab Emirates (UAE) | No rudimentary horn pregnancy |

REFERENCES

1. Jain R, Gami N, Puri M, Trivedi S. A rare case of intact rudimentary horn pregnancy presenting as hemoperitoneum. *J Hum Reprod Sci* 2010; **3**: 113–115.

2. Wang B, Zhou J, Jin H. Torsion of a rudimentary uterine horn at 22 weeks of gestation. *J Obstet Gynaecol Res* 2011; **37**: 919–920.

3. Allouche M, Le Tanguy Gac Y, Parant O. Grossesse dans une corne utérine rudimentaire : une cause rare d'hémopéritoine spontané au deuxième trimestre de grossesse. *Gynecol Obstet Fertil* 2011; **39**: e44-6.

4. Siristatidis C, Chrelias C, Kassanos D. Rudimentary-horn pregnancy: some points to review. *Arch Gynecol Obstet* 2011; **283**: 917–918.

5. Brucker SY, Rall K, Campo R, Oppelt P, Isaacson K. Treatment of congenital malformations. *Semin Reprod Med* 2011; **29**: 101–112.

6. Nadia J. Khati, Aletta A. Frazier, Kathleen A. Brindle. The Unicornuate Uterus and Its Variants.

7. Nawfal AK, Blacker CM, Strickler RC, Eisenstein D. Laparoscopic management of pregnancy in a patient with uterus didelphys, obstructed hemivagina, and ipsilateral renal agenesis. *J Minim Invasive Gynecol* 2011; **18**: 381–385.

8. Mathlouthi N, Magroun M, Slimani O, Ben Temime R, Makhlouf T, Attia L, Chahia A. Grossesse sur corne utérine rudimentaire. *Tunis Med* 2013; **91**: 415–416.

9. Köroğlu M, Kayhan A, Soylu FN, Erol B, Schmid-Tannwald C, Gürses C, Karademir İ, Ernst R, Yousuf A, Oto A. MR imaging of ectopic pregnancy with an emphasis on unusual implantation sites. *Jpn J Radiol* 2013; **31**: 75–80.

10. Lata I, Kapoor D, Agarwal S, Niyaz Z. Ruptured rudimentary horn pregnancy misdiagnosed as ruptured pseudo aneurysm internal iliac artery. *Int J Crit Illn Inj Sci* 2013; **3**: 284–285.

11. Munck DF, Markauskas A, Lamont RF, Jørgensen JS. Pregnancy in a non-communicating rudimentary uterine horn in an obese woman. *Acta Obstet Gynecol Scand* 2013; **92**: 869.

12. Torbe E, Hon M-S. Subsequent pregnancy after a ruptured rudimentary uterine horn pregnancy. *Eur J Obstet Gynecol Reprod Biol* 2013; **166**: 115.

13. Alkatout I, Honemeyer U, Strauss A, Tinelli A, Malvasi A, Jonat W, Mettler L, Schollmeyer T. Clinical diagnosis and treatment of ectopic pregnancy. *Obstet Gynecol Surv* 2013; **68**: 571–581.

14. Caserta D, Mallozzi M, Meldolesi C, Bianchi P, Moscarini M. Pregnancy in a unicornuate uterus: a case report. *J Med Case Rep* 2014; **8**: 130.

15. Akdemir A, Ergenoglu AM, Yeniel AÖ, Sendag F, Karadadaş N. Coring-type laparoscopic resection of a cavitated non-communicating rudimentary horn under hysteroscopic assistance. *J Obstet Gynaecol Res* 2014; **40**: 1950–1954.

16. Pados G, Tsolakidis D, Athanatos D, Almaloglou K, Nikolaidis N, Tarlatzis B. Reproductive and obstetric outcome after laparoscopic excision of functional, non-communicating broadly attached rudimentary horn: a case series. *Eur J Obstet Gynecol Reprod Biol* 2014; **182**: 33–37.

17. Arab M, Mehdighalb S, Khosravi D. Functional rudimentary horn as a rare cause of pelvic pain: a case report. *Iran Red Crescent Med J* 2014; **16**: e19351.

18. Takeda A, Koike W, Hayashi S, Imoto S, Nakamura H. Magnetic resonance imaging for diagnosis and management of unruptured interstitial pregnancy. *J Obstet Gynaecol Res* 2015; **41**: 1384–1393.

19. Aheri H, Saadi H, Benkirane S, Mimouni A. La grossesse gémellaire sur un utérus pseudo unicorne: à propos d'un cas. *Pan Afr Med J* 2015; **22**: 330.

20. Shiber LJ, Biscette S. Laparoscopic Management of a Rudimentary Horn Pregnancy. *J Minim Invasive Gynecol* 2015; **22**: S153.

21. Dibble EH, Lourenco AP. Imaging Unusual Pregnancy Implantations: Rare Ectopic Pregnancies and More. *AJR Am J Roentgenol* 2016; **207**: 1380–1392.

22. Le Mitouard M, Huissoud C, Fichez A, Roumieu F, Allias F, Rudigoz RC, Caloone J. Rupture utérine colmatée par l'épiploon sur grossesse développée au dépend d'une corne utérine rudimentaire : à propos d'un cas rare. *J Gynecol Obstet Biol Reprod (Paris)* 2016; **45**: 521–524.

23. Gnanasambanthan S, Uchil D. A rare case of an ectopic pregnancy in the rudimentary tube of a patient with an isolated unicornuate uterus. *BMJ Case Rep* 2017; **2017**.

24. Rutten C, Khadam L, Picamoles P, Fokou-Soh RM, Alperin E, Belaidi N. Lithopedion developed in a non-communicating rudimentary uterine horn: CT features. *Diagn Interv Imaging* 2017; **98**: 817–818.

25. Bayyarapu VB, Gundabattula SR. Diagnosis and Management of 'Cornual' Pregnancies from 2002 to 2015 at a Tertiary Referral Centre in South India: Insights from Introspection. *J Obstet Gynaecol India* 2017; **67**: 414–420.

26. Ngichabe S, Sura M. Placenta Percreta in a Gravid Bicornuate Unicollis Uterus. *Case Rep Obstet Gynecol* 2017; **2017**: 4082182.

27. Si M, Li P, Yuan Z, Ma H, Cui B, Kong B. An unexpected invasive hydatidiform mole in a rudimentary uterine horn: A case report. *Oncol Lett* 2017; **14**: 2808–2812.

28. Sánchez-Ferrer ML, Prieto-Sanchez MT, Del Sánchez Campo F. Variations in clinical presentation of unicornuate uterus with non-communicating rudimentary horn (class IIB of the American Fertility Society classification). *Taiwan J Obstet Gynecol* 2018; **57**: 110–114.

29. Zhang D-D, Gao Y, Lang J-H, Zhu L. Diagnosis and Treatment of Rudimentary Horn Pregnancy: Analysis of Eleven Cases. *Chin Med J (Engl)* 2018; **131**: 3012–3014.

30. Sawada M, Kakigano A, Matsuzaki S, Takiuchi T, Mimura K, Kumasawa K, Endo M, Ueda Y, Yoshino K, Kimura T. Obstetric outcome in patients with a unicornuate uterus after laparoscopic resection of a rudimentary horn. *J Obstet Gynaecol Res* 2018; **44**: 1080–1086.

31. Obeidat RA, Aleshawi AJ, Tashtush NA, Alsarawi H. Unicornuate uterus with a rudimentary non-communicating cavitary horn in association with VACTERL association: case report. *BMC Womens Health* 2019; **19**: 71.

32. Engku-Husna EI, Nik-Ahmad-Zuky NL, Muhammad-Nashriq K. Müllerian duct anomalies with term pregnancy: a case report. *J Med Case Rep* 2020; **14**: 209.

33. Schaible B, Haught E, Vozar A, Riggs K, Calhoun B, Bush S. Abdominal cerclage in a patient with a neocervix with planned cesarean hysterectomy at delivery. *J Obstet Gynaecol Res* 2021; **47**: 416–419.

34. Ebanga L, Dabi Y, Thomassin-Naggara I, Castaigne V, Lefebvre M, Lecarpentier E, Miailhe G, Haddad B. Approche originale en deux étapes d’une grossesse ectopique dans une corne rudimentaire : un rapport de cas unique avec une revue de la littérature. *Gynecol Obstet Fertil Senol* 2021; **49**: 943–946.

35. Coakley KE, Yang TB, Chung JH. Periviable delivery of a pregnancy in a rudimentary uterine horn: A case report. *Case Rep Womens Health* 2021; **32**: e00346.

36. Yagmurov OD, Karavaev VM, Korobkov NA, Karev VE, Tolmachev IA, Fetisov VA. Problemy ekspertnoi otsenki sluchaya fatal'nogo razryva stenki rudimentarnogo roga matki pri ektopicheskoi beremennosti. *Sud Med Ekspert* 2022; **65**: 40–45.

37. Hamet B, Hoeffel C, Fague V, Lucot J-P, Pagès-Bouic E, Rousset P, Graesslin O, Bazot M, Poncelet E. Pregnancy in a rudimentary horn: multicenter's MRI features of a rare condition. *Abdom Radiol (NY)* 2022; **47**: 4195–4204.

38. Tellum T, Bracco B, Braud LV de, Knez J, Ashton-Barnett R, Amin T, Chaggar P, Jurkovic D. Reproductive outcome in 326 women with unicornuate uterus. *Ultrasound Obstet Gynecol* 2023; **61**: 99–108.

39. Aminu MB, Sania I, Khairunnaesa M. Post-dated Breech Pregnancy in a Non-obviously Communicating Rudimentary Horn of a Bicornuate Uterus Requiring Hemi-hysterectomy. *J West Afr Coll Surg* 2023; **13**: 111–113.

40. Zheng X, Zhou Y, Sun Z, Yan T, Yang Y, Wang R. Abdominal pregnancy secondary to uterine horn pregnancy: a case report. *BMC Pregnancy Childbirth* 2023; **23**: 412.

41. Garapati J, Jajoo S, Sharma S, Cherukuri S. Unicornuate Uterus with a Non-Communicating Rudimentary Horn: Challenges and Management of a Rare Pregnancy. *Cureus* 2023; **15**: e40666.

42. Shrivastava P, Shrivastava D, Shrivastava P, Rawlani S, Chimurkar V. Rare and Occult Müllerian Deformity Diagnosed Intraoperatively During Lower Segment Caesarean Section. *Cureus* 2023; **15**: e46861.

43. Xu S, Zhang J, Yue S, Qian J, Yang L, Xu Y-Z, Zhang J. A case of rudimentary uterine horn pregnancy complicated with placental implantation in later stages of pregnancy. *Quant Imaging Med Surg* 2024; **14**: 1167–1172.

44. Srinivas T, Kirschen GW, Yazdy GM. Laparoscopic Management of a 12-Week Pregnancy Loss in a Rudimentary Uterine Horn. *Cureus* 2024; **16**: e61677.

45. Tsai S, Uzelac A, Lindheim SR, Pereira N. Surgical management of an ectopic pregnancy in the setting of an unexpected Müllerian anomaly: intraoperative and postoperative implications. *Fertil Steril* 2024; **122**: 951–953.

46. Khan SA, Molvi S, Mathew G, Khalfan M. Clinical profile and management of non-tubal ectopic pregnancy: Experience from a tertiary care hospital in the United Arab Emirates (UAE). *Pak J Med Sci* 2024; **40**: 2063–2068.
